# Variation of the Chemical Composition of Essential Oils and Total Phenols Content in Natural Populations of *Marrubium vulgare* L.

**DOI:** 10.3390/plants11050612

**Published:** 2022-02-24

**Authors:** Mounira Guedri Mkaddem, Ahlem Zrig, Mariem Ben Abdallah, Mehrez Romdhane, Mohammad K. Okla, Abdulrahman Al-Hashimi, Yasmeen A. Alwase, Momtaz Y. Hegab, Mahmoud M. Y. Madany, Abdelrahim H. A. Hassan, Gerrit T. S. Beemster, Hamada AbdElgawad

**Affiliations:** 1 Energy, Water, Environment and Process Laboratory, (LR18ES35), National Engineering School of Gabes, University of Gabes, Gabes 6072, Tunisia; mounira.mkaddem@gmail.com (M.G.M.); meriembenabdallah@yahoo.fr (M.B.A.); mehrez.romdhane@laposte.net (M.R.); 2 Faculty of Sciences of Gabès, University of Gabes, Tunisia City Erriadh, Zrig, Gabes 6072, Tunisia; 3 Department of Food Sciences & Nutrition, College of Food & Agriculture Sciences, King Saud University, Riyadh 11451, Saudi Arabia; Malokla@ksu.edu.sa (M.K.O.); aalhashimi@ksu.edu.sa (A.A.-H.); yasmeen@ksu.edu.sa (Y.A.A.); 4 Resarch Institute of Medicinal and Aromatic Plants, Beni-Suef University, Beni-Suef 62511, Egypt; momtezhegab@gmail.com; 5 Department of Botany and Microbiology, Faculty of Science, Cairo University, Giza 12613, Egypt; madany@cu.edu.eg; 6 Department of Food Safety and Technology, Faculty of Veterinary Medicine, Beni-Suef University, Beni-Suef 62511, Egypt; abdelrahimhassan@gmail.com; 7 Integrated Molecular Plant Physiology Research, Department of Biology, University of Antwerp, 2000 Antwerpen, Belgium; gerritbeemster@gmail.com (G.T.S.B.); hamada.abdelgawad@uantwerpen.be (H.A.); 8 Botany and Microbiology Department, Faculty of Science, Beni-Suef University, Beni-Suef 62511, Egypt

**Keywords:** essential oil, GC–MS, *Marrubium vulgare*, population’s structure, variability

## Abstract

*Marrubium vulgare* is a valuable source of natural bioactive molecules with high preventive and therapeutic effectiveness. Therefore, this study aimed to study the chemical polymorphism of natural populations of *M. vulgare* in Tunisia by quantitative chemical markers and the estimation of divergence between populations. Phytochemical analyses of the eight natural populations of Tunisian *Marrubium vulgare* prospected in different bioclimatic stages, revealed 42 compounds of essential oils representing 96.08% to 100% of the total oil. Hydrocarbon sesquiterpenes were the main fraction of all the populations studied and β-bisabolene was the major compound (from 30.11% to 71.35% of the total oil). The phytochemical investigation of the *M. vulgare* plant indicated the presence of essential oil with significant percentages of phenolic compounds. A significant quantitative and qualitative variation in the essential oils is detected for both major and minor compounds. The principal components analysis (PCA) performed in the single and combined traits provides a good distinction among populations, not according to their geographical and/or bioclimatic origins. Moreover, the phytochemical analysis of the leaves showed that the Tunisian populations, i.e., the populations of Kasserine, Kef, and Beja, were very rich in phenolic compounds (from 20.8 to 44.65 mg GAE/g DW). Flavonoids compounds were also the main class of total polyphenols present in all the tested populations (from 8.91 to 37.48 mg RE/g DW). The quantitative genetic diversity estimated by the population’s structure, based on PCA analysis, was an adaptation to the changes in the environmental conditions. Overall, our study indicated that natural populations of *M. vulgare* had different chemotypes of essential oils and they were rich in phenolic compounds, particularly flavonoids, which opens a new prospect for industrial use and differential exploitation of this species.

## 1. Introduction

Food flavoring, fragrance, cosmetics, and pharmaceutical companies have been in high demand for the essential oils of plants and their other secondary metabolism products in recent years, due to a rising customer interest in natural components [1]. Food, medications, and perfumes are just a few of the things for which plants have been used. They were tested to determine if they can be used to treat a range of infections and to protect foods from oxidative damage [2]. Lamiaceae species produce a great variety of secondary compounds, especially essential oils, which are found in prolruding multicelled glandular trichomes and produced in the glandular hairs on the surfaces of leaves and inflorescences [3]. In Tunisia, the Lamiaceae family is much exploited for its therapeutic virtues. In addition, about 70% to 90% of the species originate from spontaneous populations. Nevertheless, excessive exploitation of the species from this family, as for all aromatic and medicinal plants, has led to a serious reduction of population numbers and the destruction of their habitat [4]. This family is well known with two major series of genera: oil-rich and oil-poor species, and several members of the family are used as sources of essential oils. Among the species of this family, the genus *Marrubium* (Lamiaceae), includes roughly 97 species found in temperate climates, as well as the Mediterranean, Asia, America, and Australia [5]. The *Marrubium* genus grows spontaneously in all countries from north to south; it is represented by five species including *M. vulgare* L., *M. Aschersonii* P Magnus, *M. alysson*, *M. supinum* L., and *M. deserti* of Noah [6]. This plant, also known as “horehound” in Europe and “Om Rubia” in Tunisia, grows wild in dry sandy soils and wastelands, and is widely used as a raw material in the herbal extracts and beverage industries. It is also well used in traditional medicine in phytotherapy [7]. *M. vulgare* is often an important source for the food and pharmaceutical industry. For example, in Tunisia, it is well used in traditional medicine as a hypotensive, a hypoglycemia substance, and in asthma and lung diseases [8]. Tonic, aromatic, stimulant, expectorant, diaphoretic, and diuretic characteristics are all found in *M. vulgare*. Phytochemicals found within it include phenyl propanoids, phenolic compounds, and diterpenic lactones. The marrubiin is the most active diterpenoid responsible for the therapeutic properties observed from these species [9]. It displays a large variety of biological activities including antimicrobial [10], cardioprotective [11], antitumor [12], immunomodulatory [13], antioxidant [14], antidiabetic [8], antiprotozoal, gastroprotective antiviral [14], antihepatotoxic [15], vasorelaxant [16], antioedematogenic [17], insecticidal [3], and antihypertensive activities [18]. High antibacterial, antifungal, and cytotoxic properties can be explained by the high levels of essential oils in *Marrubium* species [19]. The leaves and flowering stems are used as diuretics, antispasmodics, antiseptics, and antidiabetics. Essential oils are another important secondary metabolite since they are highly bioactive in some situations, readily available in tropical countries, and commercially viable. According to Zarai et al. (2011) [20], *M. vulgare* has a moderate amount of essential oil, about 0.1%.

Limited studies on the chemical composition, and the antioxidant and antimicrobial effects of *Marrubium vulgare* L. have been investigated in Tunisia [7]. Unfortunately, in Tunisia, the natural users of this heritage (*M. vulgare*) often exploit the spontaneous populations, without interest for their durable management and their conservation. Nevertheless, the excessive exploitation and the habitat destruction of this species result in important genetic erosion, characterized by the reduction of the population size, which can lead to the decrease of gene flow levels between them. For this reason, *M. vulgare* deserves programs to safeguard the natural populations to assure their durable usage, as with the majority of other PAM, which represent a bank of natural bioactive molecules with high preventive and therapeutic effectiveness. In this regard, any politics of aromatic and medicinal genetic resources conservation necessitates a description, as exhaustive as possible, of the chemical and genetic divergence of natural populations. As far as we know, no research has been published about the genetic diversity of *M. vulgare*. Thus, the objective of the present work was to study the chemical polymorphism of natural populations of *M. vulgare* in Tunisia. Moreover, to identify the best habitat to achieve the highest antioxidant properties among the different habitat conditions. In this regard, the quantitative chemical markers (essential oil, terpene composition, total polyphenol, and flavonoid content) and the estimation of the divergence between populations could help to develop rational exploitation and conservation programs. 

## 2. Materiel and Methods

### 2.1. Plants Materials

The fresh leaves of eight populations of *M. vulgare* prospected in Tunisia in March 2018 were collected during the flowering stage. These eight populations represented most of the geographic range covered by the species in Tunisia. The characteristic of each station is defined by Emberger (1966) [21] (Table 1).

### 2.2. Essential Oil Isolation

About 250 g of fresh leaves and flowers of *M. vulgare* were separately submitted to hydrodistillation for 3 h, using a Clevenger-type apparatus (British Pharmacopoeia, 1980). Leaves were placed in 665 mL of distilled water, which represents a load report of 2/3 (0.01–0.04% yield). Anhydrous sodium sulphate was used to eliminate water traces from the essential oil. The essential oil was stored at +4 °C until tested. The condition of the culture, the extraction method, and stockage were the same for all the populations analyzed.

### 2.3. Gas Chromatography and Gas Chromatography–Mass Spectrometry

The essential oil composition was determined by a Varian Star 3400 (Les Ulis, France) Cx gas chromatograph, equipped with a flame ionisation detector (FID) and a DB-5 (30 m × 0.25 mm; film thickness 0.25 µm) capillary column. The oven temperature gradually rose from 60 °C to 260 °C, with a gradient of 5° C/min and 15 min isothermal at 260 °C, and finally rose to 340 °C at 40 °C/min. Helium (purity 99.99999%) was used as the carrier gas at 1 mL/min. The total analysis time was 57 min. A total of 1 µ of the diluted sample (1/100 in petroleum ether, *v*/*v*) was injected in the split mode (ratio 1:10). The injector was operated at 200 °C. Quantitative data were obtained electronically from the FID area percent data without the use of correction factors. Peak integration and quantification were performed automatically with Saturn 2100 Workstation software. A check of the integration of each peak was carried out and corrected manually if necessary.

Essential oil analysis was also performed with a GC chromatograph equipped with a Varian Saturn GC/MS/MS 4D mass selective detector in the electron impact mode (70 eV). MS was adjusted for an emission current of 10 µA and an electron multiplier voltage between 1400 and 1500 V. The trap temperature was 150 °C, and that of the transfer line was 170 °C. Mass scanning was from 40 to 650 amu. The components were identified based on the comparison of their Kovats indices (KI), co-injection of standards, and MS experimental data, with those contained in commercial or literature libraries (NIST 02 version 2.62, Adams, 2001) [22]. Alkanes (C5–C24) were used as reference points in the calculation of KI. GC and GC–MS analysis results are given in Table 2. All the determinations were performed in duplicate and averaged.

### 2.4. Determination of Bioactive Compounds

#### 2.4.1. Extract Preparation

A total of 2 g of leaf powder was crushed and extracted with 20 mL of methanol for 24 h at ambient temperature and in darkness. After filtration and removal of methanol under reduced pressure in a rotary evaporator, the dry residue was dissolved in absolute methanol (1 g of extract in 10 mL of methanol) for conservation and analysis.

#### 2.4.2. Quantity Composition of Polyphenol

The dosage of total polyphenols was determined with Folin–Ciocalteu reagent using the method of Mau et al. (2001) [23]. The absorbance measured at λ = 760 nm with a UV-visible spectrophotometer is proportional to the quantity of polyphenol present in the plant extracts. A total of 1 mL of the diluted samples was mixed with 1 mL of Folin reagent and 4 mL of ultra-pure water. After incubation for 3 min at ambient temperature, 10 mL of sodium carbonate solution, Na_2_CO_3_ (1 M), was added. This mixture was adjusted to a volume of 20 mL by the ultra-pure water. The mixtures were incubated for 90 min. To ensure the reliability of the results, the dosage of each phenolic compound was realized in three tests.

The phenol standard used is the Gallic acid. The levels of total polyphenols were expressed as the milligram gallic acid equivalents (GAE) per gram of dry weight (mg GAE/g DW).

#### 2.4.3. Total Flavonoids Content

The determination of flavonoids concentration was carried out by Djeridane et al. (2006) [24]. The method is based on the formation of a complex aluminum-flavonoid with a yellow color in the presence of the soda. This staining absorbs in the visible range at 430 nm. A total of 1 mL of each appropriately diluted extract or standard solution of rutin was mixed with 1 mL of aluminum trichloride solution, AlCl_3_ (0.3 M); the mixtures were incubated for 15 min. The concentrations of flavonoids were expressed as the milligram rutin equivalent per gram of dry weight (mg RE/g DW). Samples were analyzed in triplicate.

#### 2.4.4. HPLC Analysis of Phenolic Profile

The HPLC system consisted of a RP-HPLC (serie 1200, AGILENT TECHNOLOGIES) series instrument, coupled to a quaternary pump, a diode array detector (DAD), an autosampler and a column compartment. UV–visible spectra were recorded from 210 to 520 nm and chromatograms were monitored at 280 nm, 320 nm, and 360 nm. Samples were separated on a Spherisorb C18 (5 m, 250 × 4.6 mm) column, with a sample injection volume of 0.01 mL. The mobile phase consisted of water–formic acid (100:0.1, *v*/*v*) (A) and MeOH–formic acid (100:0.1, *v*/*v*) (B). The eluting conditions applied were: 0–5 min, linear gradient from 10% to 20% B; 5–15 min, linear gradient from 20% to 40% B; 15–45 min, linear gradient from 40% to 100% B, for washing return to 10% B at 50 min, and finally 5 min isocratic to reequilibrate the column. The mobile phase flow rate was 0.7 mL/min and the column temperature was controlled at 30 °C [24].

## 3. Statistical Methods

The results were presented as the means ± SEM of the triplicate measurements. To better compare the means of the characters between populations, we conducted one-way analysis of variance (ANOVA). The means of the populations were the mean of the overall observations, allowing for defining the gap between the different populations. A comparison of the means by the Duncun test was also performed (SAS software).

To better estimate the divergence between the populations based on the chemical markers, we conducted principal components analysis (PCA) (SAS, 2006) [25]. This was based on terpenes, phenols, and flavonoids content, and all parameters conjoined. Correlation analysis between all the parameters was also carried out using the statistical correlation test (SAS, 2006).

## 4. Results and Discussion

### 4.1. Qualitative Analysis of Essential Oil Composition and Population Structure

The components of the essential oils were identified by their percentage and their retention indices (RI). Table 2 presents the components with a percentage above 0.1 in order of their elution on the HP-5 column. The components that are present in a percentage lower than 0.1, or as trace, are not listed. The composition of the oil varies between the different locations. Analysis of *M. vulgare* essential oil revealed the presence of 42 compounds which represents 96.08–100% of the total oil (Table 2). The hydrocarbon sesquiterpenes are the main fraction in all the populations tested, and the highest percentage was recorded in the Zagouan population. β-bisabolene is the major constituent, with levels ranging from 32% in population Beja (**1**) to 65.2% in population Zagouan (**5**). 

Furthermore, β-bisabolene has a balsamic odor and is approved in Europe as a food additive. β- and γ-bisabolenes have been found to possess anticancer properties [26]. The present study revealed that the population of Kasserine had benzodioxole (phenols) as their major component (50.14%). The second major compound was germacrene D (sesquiterpene hydrocarbons), with levels varying from trace in P2, P5, and P7, to 17.05% in population Nabeul (**3**). Terpinen, camphor, borneol, and α-terpineol were present only in the population Beja (**1**). The β-caryophyllene (sesquiterpene hydrocarbons) was frequently shared between different populations except population **7**. On the other hand, eudesmol and isocaryophyllene were found only in population Gabes (**8**), and benzodioxole and *trans*-isodillapiole (phenol) were present only in the population of Kasserine (**7**) (Table 2). 

Importantly, sesquiterpene hydrocarbons represent the main fraction of the oil, this was in line with the chemical composition of *M. vulgare* grown in Ouled Mnasser Djebeniana, Tunisia [20]. It is interesting to note that the presence of benzodioxole (a phenylpropanoids derivative) in population **7** (Kasserine), as a major compound with 50.14% of the total oil, has never been found in the essential oils of the *Marrubium* genre or Lamiaceae family. A similar result was elucidated in the young roots and aerial parts of *Astrodaucus persicus* (Apiaceae) extract [27]. Benzodioxoles are important in health and used as antitumor, antibacterial, antifungal, antiparasitic, antimalaria, and antioxidant drugs, as well as in pesticides and herbicides [28]. This population also contains a high proportion of *trans*-isodillapiole (19.07%) (a phenylpropanoids derivative). This specific composition could probably be due to an adaptation of *M. vulgare* to the bioclimatic conditions of the region or to intrinsic factors related to the genetics of the plant. Kadri et al. (2011) [7] also found that the major compound of *M. vulgare* essential oil is eudesmol. However, this compound was only detected in the essential oil of population Beja (**5**). The more abundant chemical component in Boussalem and Tunis is eugenol [29]. Furthermore, the citronellol and lemonellyl format was not detected in all the extracts. β-bisabolene is a major component of the essential oil of *M. vulgare* from Iran [30]. Khanalvi et al. (2005) [31] have found that the major constituent of *M. vulgare* from Turkey is β-bisabolene. Although Asadipour et al. (2005) [32] have revealed that caryophyllene oxide, caryophyllene, and germacrene D are the main fractions. The main compound of *M. vulgare* oil from Poland was caryophyllene [33]. Nagy and Svajdlenka (1998) [34] revealed that the major constituents of *M. vulgare* essential oil from the Czech Republic are caryophyllene and germacrene D. However, Weel et al. (1990) [35] have identified Z-Farnesene, caryophyllene, (E)-2 hexanal, and α-humulene, as new chemotypes of Lithuanian *M. vulgare*. A specific composition with the presence of chemical components that are not identified as the usual constituents of *M. vulgare* essential oil in other parts of the world (significant percentages of the phenolic compounds, fatty acids, and alkanes) is revealed. A quantitative and qualitative variation concerns both minor and major compounds, indicating that the environmental factors strongly influence its chemical composition (Table 2). 

**Figure 1:**
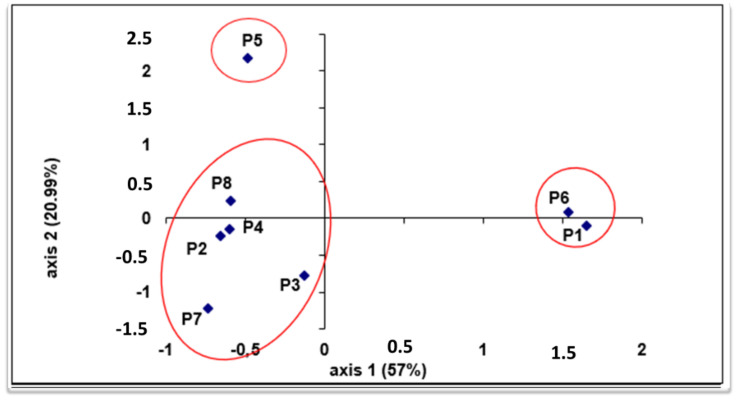
Principal components analysis (PCA) performed on the major essential oil component for the eight *M. vulgare* populations analyzed. Plots according to the two axes, 1–2. Numbers indicates the populations (P1: Beja, P2: Bizerte, P3: Nabeul, P4: Sousse, P5: Zagouan, P6: Kef, P7: Kasserine, P8: Gabes).

The chemical structure of the populations by principal components analysis (PCA) performed on the terpene markers, most present and shared between populations, shows that the first two PCA axes represent 78% of the total variation (Figure 1). The populations were clearly isolated and subdivided into four distinct groups (Figure 1), but this separation was not based on their geographical and/or bioclimatic origins. Accordingly, the observed wide variation could not only be explained by climate variation because there were both quantitative and qualitative variations of terpenic compounds within the same ecological group, and between the geographical locations of the populations. Some authors report that high chemical divergence was not correlated with geographic distance with regard to the structuration of many other medicinal and aromatic plants [36,37]. Significant differences in the chemical compounds of the essential oils have been found between geographically near populations (Nabeul, Sousse, and Zagouan or Kef and Kasserine). 

Additionally, this observed terpenic variability between *M. vulgare* populations can be explained in part by the variation of environment parameters that, defined by Emberger (1966) [21], include annual rainfall and the difference between the maximum temperatures of the warmest months and the minimum temperatures of the coldest months. Although various other environmental factors (topography, altitude, seasonal distribution of precipitation, etc.) would have a great deal to do with the variation of the chemical compounds in essential oil [38,39,40]. Additionally, chemical variability could be explained by local abiotic factors characteristic of the scaling zone and/or selective biotic factors (fauna and flora) [37]. Edaphic conditions also play an important role in the modification levels of these compounds [4]. It should also be noted that the geographical area of all Mediterranean countries, including Tunisia, is characterized by variable reliefs including long mountain ranges relatively close to the sea. As a result, climatic differences exist between the neighboring regions, mainly due to differences in altitude reflected in the differentiation of local bioclimate [37,38]. However, the variation in terpene levels between populations may also result from the influence of plant-specific genetic factors, that are related to the plant’s reproductive patterns [41,42]. Additionally, the effect of genotype-middle interaction can play an important role in this variation. Considering this conclusion, it is worth noting that there are significant qualitative and quantitative variances in essential oils among populations, implying that environmental influences have a significant impact on their chemical composition.

### 4.2. Analysis of Total Polyphenol

**Figure 2:**
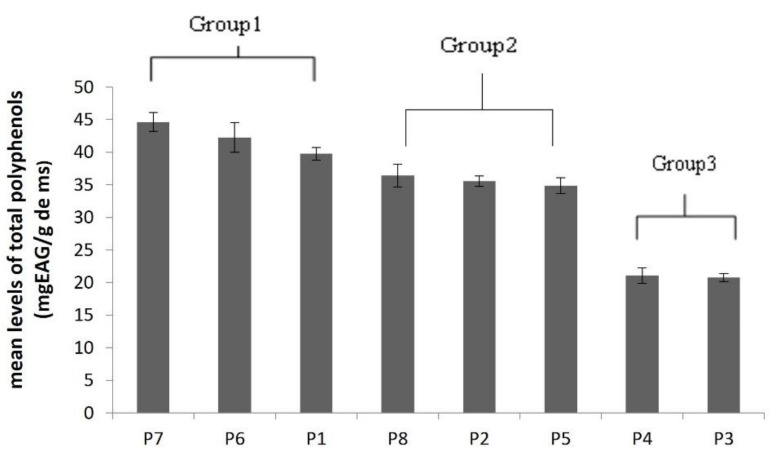
Changes in the total polyphenols in the leaves of the eight *M. vulgare* L. populations.

Polyphenolic substances are the key chemical groups that have the potential to remove radical species, inhibiting the development of oxidative chain reactions, and functioning as primary antioxidants or free radical terminators [43], hence, quantifying polyphenols in various samples is critical. Plants’ phenolic composition and content vary widely due to genetics and environmental factors [44]. Numerous studies [8,45,46] have shown that intraspecific diversity amongst populations from various locations can lead to differences in antioxidant properties. Our results showed that the quantification of secondary metabolites from *Marrubium vulgare*, which represents a natural antioxidants source, indicated that the highest amount of phenolic compound was found in the methanolic extract of the Kasserine and Kef populations (44.65 and 42.16 mg GAE/g DW, respectively). However, the lowest amount was found in Nabeul’s methanolic extract, with a content of 20.80 mg of GAE/g DW (Table 3). Analysis of the variance to one criteria of classification (population effect) on the average of the total polyphenol levels shows highly significant variations between populations (*p* > 0.01) (Table 3). This significant variation between populations may be due to the variation in environmental conditions (climate, pressure, humidity and edaphic) [47,48], while the polyphenols are involved in the adaptation of species to the bioecological conditions [49,50]. The Duncun test is used to separate the population into three groups, i.e., populations **7**, **6** and **1** form the first group, while populations **8**, **2** and **5** compose the second group. However, populations **4** and **3** are agglomerated in a third group (Figure 1). Our result is more important than the levels detected in the *M. vulgare* from other origins in Tunisia [29], and it is different from those obtained from *M. vulgare* in Poland [51]. However, this agglomeration does not conform with the bioclimatic characteristics of the populations (Figure 2). In fact, populations from inferior humid bioclimates (Beja and Bizerte) have very different total phenolic contents, and rearranging them into different groups with Duncun test analysis, indicated that edaphic conditions are one of the major factors influencing the differences in the chemical composition of metabolites and bioactivities in plants. Other researchers in Tunisia species demonstrate substantial differences in the total polyphenols content and explained this variability by edaphic characteristics, especially soil salinity [52].

### 4.3. Individual Phenolic Acids Compounds in M. vulgare

*M. vulgare* is an abundant source of various phenolic compounds, major classes being phenolics acids and flavonoids. The composition of the identified compounds presented eight compounds in eight populations. We identified ferulic, quercetin, p-coumaric and caffeic acids as the main detected flavonoid and phenolic acids (Table 4). Our results are in agreement with those reported for Polish *M. vulgare*, where ferulic acid (36.2 mg/100 g dry matter), luteolin (616 mg/100 g dry matter) and apigenin (43.8 mg/100 g dry matter) were the major phenolic compounds [12]. In the present study ferulic acid is the main phenolic compound in all the populations, except Kef, where the highest amount was recorded in catechin, which occurs as the main constituent of *M. vulgare*. It is worth noting that phenolic acid yields depend on the leaf sampling locality. Indeed, the best yield of ferulic acid was observed for the Bizert extracts, followed by those of Beja and Kasserine, respectively. These changes can be explained by the climatic and edaphic differences characterizing each geographical region. The antioxidant properties of phenolics are associated with phenolic hydroxyl groups connected with ring structures [30]. Interestingly, the antioxidative properties of *M. vulgare* were associated with hepatoprotective [31] and anticancer activity [32].

### 4.4. Analysis of Flavonoids Composition

**Figure 3:**
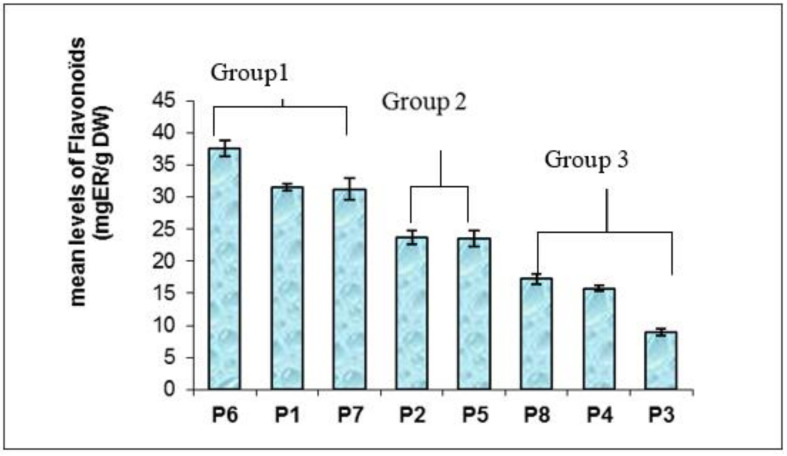
Changes in the total flavonoids in leaves of the eight *M. vulgare* L. populations.

Flavonoids are the main antioxidant components; they are a class of polyphenols [52]. The highest level of flavonoids was revealed in the methanolic extract of Kef (37.48 mg RE/g DW) (Table 3). However, the lowest amount was found in Nabeul’s methanolic extract, with a content of 8.91 mg RE/g DW. Our results confirm the findings by Amri et al. (2017) [53], which revealed a high content of phenols and flavonoids with colorimetric assays, in methanolic extracts of *M. vulgare* in Tunisia. In the literature, *M. vulgare* is reported to be rich in flavonoids [54]. However, the values detected for other populations are relatively higher (Bizert, Beja, Zagouan, Kef, Kasserine and Gabes) (Table 3). Analysis of the variance to one criteria of classification (population effect) on the average of the flavonoid levels shows highly significant variations for all the tested populations (*p* > 0.01). For the Duncun test there are three groups of populations: population **1**, **6** and **7** form the first group; **2** and **5** compose the second group, however, populations **3**, **4** and **8** are regrouped together in a third group (Figure 3).

### 4.5. Structure of Population Based on Polyphenols and Flavonoids Content

The principal components analysis (PCA) performed on the total polyphenol and flavonoids levels established according to the first two principal components (100% of the total variation, Figure 3), revealed that population groupings in three agglomerations, not pertaining to their bioclimate. Climate variation alone was unable to explain the chemical heterogeneity between the populations.

A correlation test between polyphenols and flavonoids levels shows that there is a positive and highly significant correlation (r = 0.853, *n* = 24, *p* < 0.0001). Populations rich in total polyphenols generally have the highest levels of flavonoids, including the population of Kasserine, Kef, and Beja. (Table 3). Moreover, the populations with low concentrations of polyphenols also have low levels of flavonoids: Sousse and Nabeul. This agglomeration confirmed that obtained with the Duncun test.

### 4.6. PCA of Combined Analysis

**Figure 4:**
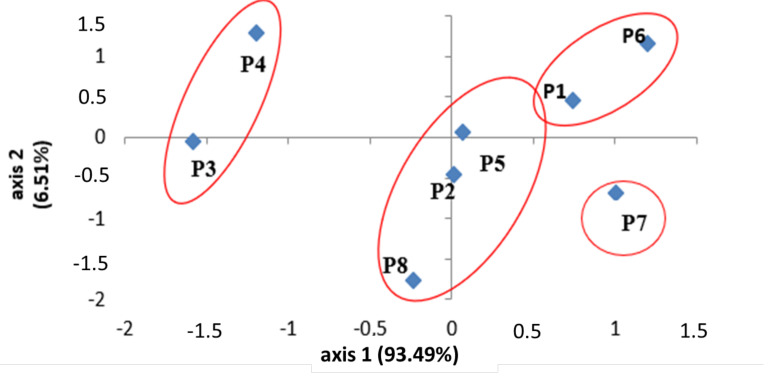
Principal components analysis (PCA) performed on the levels of polyphenol and flavonoids content for the eight *M. vulgare* L. populations analyzed. Plots according to axis 1–2. Number indicates the populations (P1: Beja, P2: Bizerte, P3: Nabeul, P4: Sousse, P5: Zagouan, P6: Kef, P7: Kasserine, P8: Gabes).

**Figure 5:**
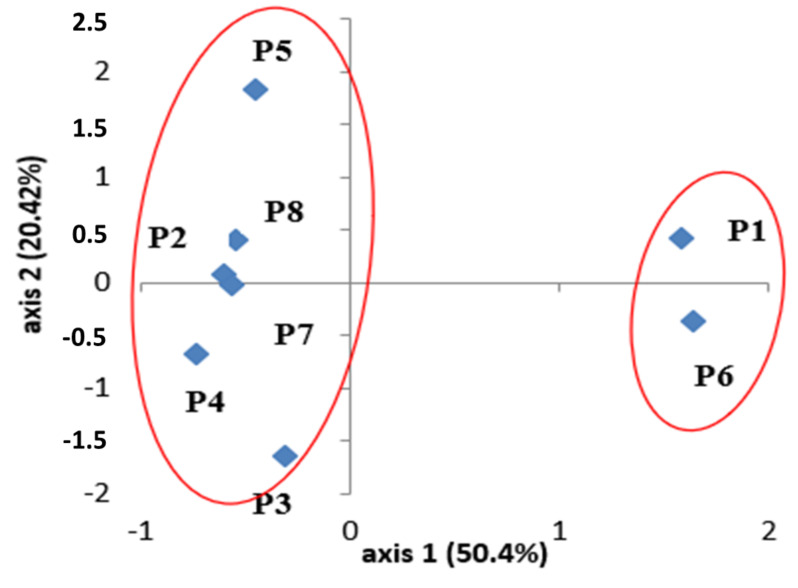
Principal components analysis (PCA) performed on essential oil component polyphenol and flavonoids markers combined for the eight *M. vulgare* L. populations analyzed. Plots according to the two axes 1–2. Numbers indicate the populations (P1: Beja, P2: Bizerte, P3: Nabeul, P4: Sousse, P5: Zaouan, P6: Kef, P7: Kasserine, P8: Gabès).

PCA is a multivariate statistical analysis that can be used to examine and simplify complex and huge datasets (Figure 4 and Figure 5). The pattern of variation in *M. vulgare* was also analyzed using principal components analysis (PCA) to evaluate the species and their link with the observed traits, based on the correlation between the traits and the extracted clusters. The chemical profiles of plants that species were grouped and clearly separated from the populations. The joint analysis by principal components analysis (PCA), based on phenolic and terpenic markers combined, showed that the chemical composition of the populations was influenced by these two quantitative markers. Indeed, there was no change in the profile of the terpene compounds of populations **1**, **5**, **6** and **8**, and that was explained by the fact that populations **1** and **6** both have high levels of terpene compounds (most correlated to PC 1) and high total polyphenols. Population **5** has both higher and lower levels of phenolic compounds, and high levels of total polyphenols. Population **8** has moderate levels of terpene compounds and phenolic compounds. Compared with PCA only performed for phenolic compounds, populations **3** and **4** showed different locations, according to their low phenolic content; these populations also had average levels of terpenes. The population of Kasserine (**7**) showed that the impact of climate variation alone could not explain this chemical variability. It can, therefore, be concluded that the quantitative determinism of the genes encoding these adaptive traits (the secondary metabolites) was complex and was controlled by various environmental and plant genetics factors.

All the results on the chemical structuring of populations show that chemical polymorphism between natural populations of *M. vulgare* in Tunisia was very important, regardless of the used marker. They also show that the determinism of quantitative genes encoding these adaptive traits was influenced by several factors. This complexity was due to the importance of the plant genotype effect, which was the first assuring biosynthesis of these biomolecules, and to the importance of the effect of genotype-middle interaction, that regulates the expression of genes encoding these secondary metabolites, while also noteworthy is the major effect exerted by the environment, such as the climate, soil texture, pathogens, etc.

## 5. Conclusions

*M. vulgare* from Tunisia is a chemotype of β-bisabolene. A significant quantitative and qualitative variation in the chemical compounds of the essential oils is detected. This chemical divergence concerns both major and minor compounds. *M vulgare* populations are very rich in total polyphenols and flavonoids. The chemical structuring of populations by PCA, based on the most present and shared terpene markers, and the total polyphenol and flavonoids levels separately, is not necessarily explained by the populations’ bioclimatic or geographic characteristics. This also reflects that climate variation could not explain the observed wide chemical variation. Moreover, the joint action of all the factors (terpenes, polyphenols, and flavonoids) defines the quantitative genetic diversity. The latter seems to be very abundant in the populations of *M. vulgare* in Tunisia. This quantitative genetic diversity, probably controlled by polymorphic genes, is an adaptation strategy to the changes in the environmental conditions. Overall, this form of genetic diversity plays a very important role as a solution to the problems that threaten the species.

## Figures and Tables

**Table 1:** Geographic localization and bioclimatic characteristics of different Tunisian *M. vulgare* L. populations.

Populations	Code	Bioclimatic Zone	Variant Climatic	Rainfall (mm/year)	Altitude	Longitude	Latitude
**Emdjez elbeb (Beja)**	**1**	Sub-humid	Hivers doux	640	480	36°74′	9°14′
**Sidi Othmen (Bizerte)**	**2**	Sub-humid	Hivers doux	610	508	37°16′	9°65′
**Hammamet (Nabeul)**	**3**	Inferior semi-arid	Hivers doux	300–400	90	36°4′	10°58′
**Bouficha (Sousse)**	**4**	Inferior semi-arid	Hivers doux	339	25	35°83′	10°64′
**Zagouan**	**5**	Higher semi-arid	Hivers frais	527	390	36°4′	10°14′
**Kef**	**6**	Inferior semi-arid	Hivers frais	400–500	350	36°17′	8°7′
**Tala (Kasserine)**	**7**	Inferior semi-arid	Hivers frais	130–200	966	35°34′	8°40′
**Matmata (Gabes)**	**8**	Arid	Hivers doux	100–150	354	10°04′	33°28′

Bioclimatic zones are defined by Emberger’s pluviothermic coefficient: Q2 = 2000 P/(M2–m^2^), where P is the average annual rainfall (mm), M is the mean maximum temperature (K) for the warmest month, and m is the mean minimum temperature (K) for the coldest month. P, M, and m are calculated as the average for the period from 1953 to 2003.

**Table 2:** Chemical composition (% of the total essential oil) of essential oil from leaves of the eight *M. vulgare* L. populations.

Compounds	RT ^a^	IK ^b^	P1	P2	P3	P4	P5	P6	P7	P8	Mean ^c^ ± SD
δ-terpinene	6.26	1057	0.18 ± 0.003	-	-	-	-	-	-	-	0.04 ± 0.0003
Camphor	7.64	1143	1.08 ± 0.02	-	-	-	-	-	-	-	0.07 ± 0.002
Borneol	7.96	1166	0.82 ± 0.03	-	-	-	-	-	-	-	0.06 ± 0.003
α-terpineol	8.10	1185	1.56 ± 0.005	-	-	-	-	-	-	-	0.22 ± 0.0006
Pentadecanone	8.89	1671	-	-	17.05 ± 0.01	-	-	-	-	-	1.90 ± 0.001 **
Bornyl Acetate	9.61	1285	3.01 ± 0.04 a	-	-	-	-	-	-	0.4 ± 0.1 b	0.45 ± 0.01
α-copaene	10.86	1388	2.7 ± 0.07 a	-	-	-	-	3.22 ± 0.0 a	-	-	0.70 ± 0.01
β-elemene	11.06	1389	3.01 ± 0.03 b	-	-	-	-	6.14 ± 0.05 a	-	-	1.13 ± 0.01
Isocaryophyllene	11.29	1414	-	-	-	-	-	-	-	3.71 ± 0.02	0.43 ± 0.002
**β-Caryophyllene**	11.47	1415	7.26 ± 0.015 b	9.51 ± 0.18 a	7.4 ± 0.5 b	6.01 ± 0.0 b	3.74 ± 0.1 c	9.4 ± 0.2 a	-	3.07 ± 0.0 b	4.93 ± 0.2 **
α-humulene	11.92	1454	3.04 ± 0.079 a	-	-	-	-	-	-	2.05 ± 0.01 a	0.73 ± 0.002
**Germacrene D**	12.26	1480	14.57 ± 0.24 a	-	17.05 ± 0.04 a	7.62 ± 0.01 c	-	11.2 ± 0.1 b	-	-	10.51 ± 0.2 **
*Trans*-β-ionone	12.28	1534	-	-	-	-	-	-	-	6.2 ± 0.5	0.72 ± 0.031
**β-Bisabolene**	12.50	1544	32 ± 1.90 b	32.1 ± 0.91 b	32.21 ± 0.01 b	38.13 ± 0.1 b	65.2 ± 0.3 a	27.8 ± 0.70 c	2.32 ± 0.02 d	46.1 ± 0.08 e	38.21 ± 0.50 **
δ-cadinene	12.72	1559	7.773 ± 0.94 b	-	2.08 ± 0.028 b	-	-	12.8 ± 1.2 a	-	5.2 ± 0.0	5.1 ± 0.3
8-Epi-11-Nordriman-9one	13.24	1614	-	-	-	-	-	-	-	2.9 ± 0.01	0.20 ± 0.001
Vulgarol B	13.24	1688	-	-	-	-	-	-	-	2.12 ± 0.02	0.30 ± 0.002
β-*Trans* isolimonene	13.33	983	-	-	-	-	-	-	-	2.01 ± 0.06	0.27 ± 0.007
Camphene	13.34	954	1.67 ± 0.06	-	-	-	-	-	-	-	0.14 ± 0.007
Naphtalene	14.73	1179	-	8.16 ± 0.3 b	-	14.401 ± 0.02 a	-	-	-	-	2.02 ± 0.04 **
Thunbergol	14.28	2032	-	7.01 ± 0.1 a	-	-	-	5.3 ± 0.21 a	-	-	1.5 ± 0.038
β-H-Pregna	14.76	2061	-	-	-	-	-	-	2.02 ± 0.02	-	0.31 ± 0.002
Junipene	14.82	1555	-	-	-	-	-	-	13.02 ± 0.7	-	1.2 ± 0.01 **
Caryophyllene oxyde	14.28	1580	2.59 ± 0.12 b	-	-	-	5.07 ± 0.2 a	4.04 ± 0.04 a	-	-	1.3 ± 0.06
α-Eudesmol	14.37	1650	1.38 ± 0.02	-	-	-	-	-	-	-	0.22 ± 0.002
Cedrenol	14.37	1604	-	-	-	-	-	-	-	2.5 ± 0.01	0.32 ± 0.001
Phenol-2-methoxy-4propenyl	10.69	2250	-	-	-	-	-	-	-	3.0 ± 0.06	0.24 ± 0.007
**Benzodioxole**	13.02	1530	-	-	-	-	-	-	50.14 ± 0.4	-	8.6 ± 0.017
Imidazole	13.56	1055	-	-	-	-	-	-	-	5.58 ± 0.1	0.39 ± 0.8
*Trans*-Isodillapiole	13.65	1706	-	-	-	-	-	-	24.2 ± 0.5	-	3.08 ± 0.1 **
3-buten-2-ol-benzoate	14.29	1036	9.08 ± 0.2 a	-	3.5 ± 0.2 b	5.05 ± 0.05 b	-	-	-	-	1.70 ± 0.056
Benzenedicarboxilic acid	14.50		-	-	6.7 ± 0.21 b	16.01 ± 0.02 a	-	-	-	-	3.02 ± 0.2 **
Ethanoate	15.39	807	1.84 ± 0.01 b	-	-	7.04 ± 0.01 a	-	-	-	-	1.27 ± 0.002
Hexadecanoic acid	14.62	1984	-	17.02 ± 0.8 a	-	-	13.33 ± 0.7 b	-	-	-	3.70 ± 0.6 **
Tridecanoic acid	14.66	1746	-	13.11 ± 0.2	-	-	-	-	-	-	1.05 ± 0.01 **
Octadecanoic acid	14.66	2240	-	-	-	-	12.6 ± 0.1 a	-	12.6 ± 0. 3 b	-	3 ± 0.02 **
Tetradecanoic acid	14.69	2275	1.74 ± 0.01	-	-	-	-	-	-	-	0.21 ± 0.001
Propane	14.83	810	-	-	7.01 ± 0.3	-	-	-	-	-	1.09 ± 0.1 **
Hexadecane	11.80	1600	-	13.09 ± 0.3 a	7.0 ± 0.25 b	-	-	6.26 ± 0.3 b	-	9.07 ± 0.7 b	4.02 ± 0.3 **
Decane	11.81	999	5.23 ± 0.441 b	-	-	5.76 ± 0.04 b	-	14.28 ± 0.8 a	-	-	2.45 ± 0.1 **
Hexavinyldisilethylene	15.39	2076	-	-	-	-	-	-	-	4.99 ± 0.05	0.67 ± 0.006
% Alcane			45.23 ± 0.11	13.09 ± 0.075	14.01 ± 0.137	45.76 ± 0.01	-	20.54 ± 0.275	-	14.06 ± 0.187	8.23 ± 0.126
% Monoterpenes hydrocarbons			1.85 ± 0.015	8.16 ± 0.075	-	14.01 ± 0.005	-	-	-	2.401 ± 0.015	2.47 ± 0.01
% Monoterpenes oxygenated			6.47 ± 0.019	-	-	-	-	-	-	6.6 ± 0.12	7.45 ± 0.008
% Diterpenes			-	7.01 ± 0.033	-	-	-	5.43 ± 0.07	-	5.02 ± 0.01	1 ± 0.014
% Sesquiterpenes hydrocarbons			70.75 ± 0.327	41.61 ± 0.108	58.74 ± 0.578	51.76 ± 0.011	68.94 ± 0.04	67.02 ± 0.225	17.36 ± 0.074	26.33 ± 0.09	63.24 ± 0.071
% Sesquiterpenes oxygenated			3.97 ± 0.125	-	17.05 ± 0.002	-	5.07 ± 0.05	4.04 ± 0.01	-	2.50.002	3.43 ± 0.015
% Phenolics			10.92 ± 0.03	-	10.4 ± 0.058	26.06 ± 0.011	-	-	71.6 ± 0.128	48.58 ± 0.22	18.3 ± 0.16
% Fatty Acid			1.74 ± 0.002	20.13 ± 0.25	-	-	13.90 ± 0.2	-	12.6 ± 0.075	-	7.90 ± 0.157
Yields of extraction (%)			0.02	0.02	0.018	0.015	0.04	0.03	0.037	0.025	0.021

^a^: Retention time (mn) on a HP-5MS nonpolar column relative to C8–C24, ^b^: Retention index, -: Not determined, P1: Beja, P2: Bizerte, P3: Nabeul, P4: Sousse, P5: Zagouan, P6: Kef, P7: Kasserine, P8: Gabes. (**) highly significant at *p* < 0.001 with F-test of the variance analysis. Values followed by the same letter are not significantly different according to the Duncun test at *p* > 0.05; ^c^: mean at species level.

**Table 3:** Total polyphenol and flavonoid levels in leaves of the eight *M. vulgare* L. populations. The results are expressed in mg of gallic acid equivalent/g of dry weight (mg GAE/g DW) for polyphenols and mg rutin equivalent/g of dry weight for flavonoids.

Code	Populations	Total Polyphenols (mg GAE/g DW)	Total Flavonoids (mg RE/g DW)
**1**	**Beja**	39.77 ± 0.985 a	31.53 ± 0.471 a
**2**	**Bizerte**	35.56 ± 0.819 b	22.62 ± 1.114 b
**3**	**Nabeul**	20.80 ± 0.602 c	8.91 ± 0.537 c
**4**	**Sousse**	21.10 ± 1.180 c	15.75 ± 0.461 c
**5**	**Zagouan**	34.86 ± 1.204 b	24.43 ± 1.306 b
**6**	**Kef**	42.16 ± 2.29 a	37.48 ± 1.266 a
**7**	**Kasserine**	44.65 ± 1.46 a	31.20 ± 1.66 a
**8**	**Gabes**	36.42 ± 1.73 b	17.20 ± 0.789 c

Values are given as the mean ± SD (*n* = 3). Values in each column followed by different letters are significantly different (*p* < 0.05).

**Table 4:** Evaluation of the most abundant phenolic acids and flavonoids (µg/g dry weight) detected in *Marrubium vulgare* extract obtained from eight populations. Values are given as the mean ± SD (*n* = 3). Values in each column followed by different letters are significantly different (*p* < 0.05).

	Caffeic Acid	Ferulic Acid	Protocatechuic Acid	Catechin	p-Coumaric Acid	Syringic Acid	Quercetin	Myricetin	Apigenin	Luteolin	Rutin	Ellagic Acid	Eugenol
**Beja**	0.83 ± 0.01 b	31.622 ± 6.077 c	16.779 ± 3.955 c	6.543 ± 2.099 c	17.849 c ± 5.911	8.647 ± 1.265 d	23.780 ± 4.790 b	0.741 ± 0.106 b	2.313 ± 0.332 ab	3.706 ± 0.746 c	16.888 ± 3.617 b	0.090 ± 0.010 c	0.069 ± 0.005 c
**Bizerte**	0.66 ± 0.05 c	50.222 ± 3.618 a	24448 ± 4.474 a	9.323 ± 0.579 b	22.446 ± 6.261 b	13.973 ± 1.266 c	33.896 ± 6.357 a	1.023 ± 0.221 a	3.100 ± 0.689 a	5.141 ± 0.948 b	23.442 ± 4.171 a	0.122 ± 0.038 b	0.095 ± 0.036 b
**Nabeul**	0.32 ± 0.11 d	24.782 ± 1.824 c	12.115 ± 2.113 c	4.623 ± 0.247 c	12.589 ± 0.735 c	7.011 ± 0.556 d	14.242 ± 4.488 c	0.445 ± 0.139 c	1.391 ± 0.433 b	2.220 ± 0.699 c	10.106 ± 3.204 c	0.055 ± 0.017	0.042 ± 0.014 d
**Sousse**	0.44 ± 0.04 d	25.583 ± 0.959 c	14.083 ± 2.526 c	4.888 ± 0.080 c	13.192 ± 0.195 c	7.205 ± 0.329 d	17.522 ± 8.178 c	0.530 ± 0.225 c	1.654 ± 0.701 b	2.731 ± 1.275 c	12.521 ± 5.958 c	0.064 ± 0.025	0.047 ± 0.016 d
**Zagouan**	0.43 ± 0.01 d	42.038 ± 0,302 b	20.300 ± 4.438 a	8779 ± 0.477 b	24.150 ± 3.858 a	13.750 ± 1.521 b	20.624 ± 0.600 b	0.686 ± 0.063 bc	2.143 ± 0.178	7.500 ± 0.107 b	14.437 ± 0.171 bc	0.140 ± 0.042 b	0.098 ± 0.028 b
**Kef**	1.51 ± 0.05 a	18.153 ± 3.630 d	6.300 ± 0.280 d	145.320 ± 14.131 a	0.140 ± 0.000 d	17.080 ± 2.461 a	1.493 ± 0.081 d	1.633 ± 0.081 a	2.660 ± 0.370 ab	12.180 ± 1.960 a	4.573 ± 0.820 d	0.056 ± 0.000	0.047 ± 0.008 d
**Kasserine**	0.47 ± 0.01 d	45.506 ± 0.992 b	23.764 ± 2.284 ab	9.341 ± 0.436 b	24.704 ± 2.675 a	15.516 ± 0.686 ab	32.361 ± 9.275 a	1.151 ± 0.390 a	3.258 ± 0.852 a	11.868 ± 3.481 a	22.876 ± 6.735 a	0.266 ± 0.042 a	0.182 ± 0.028 a
**Gabes**	0.46 ± 0.01 d	44.971 ± 0.328 b	31.667 ± 4.817 a	8.492 ± 0.518 b	17.837 ± 4.187 c	18.225 ± 1.651 a	21.084 ± 0.651 b	0.881 ± 0.068 b	1.940 ± 0.193 b	7.908 ± 0.116 b	15.298 ± 0.186 b	0.392 ± 0.042 a	0.266 a ± 0.028
**RT (mn)**	20.500	26.400	27.800	21.500	25.100	21.700	36.870	34.270	39.450	29.450	30.610	39.400	37.400

## Data Availability

Not applicable.

## References

[1] Akther N., Shawl A.S., Sultana S., Chandan B.K., Akhter M. (2013). Hepatoprotective activity of *Marrubium vulgare* against paracetamol induced toxicity. J. Pharm. Res..

[2] de Olivera P.A., Santin J.R., Lemos M., Klein L.C.J., Couto A.G., Bittencourt C.M.S., Cechinel F., Valdir F.A. (2011). Gastroprotective activity of methanol extract and marrubiin obtained from leaves of *Marrubium vulgare* L. (Lamiaceae). J. Pharm. Pharmacol..

[3] El Bardai S., Lyoussi B., Wibo M., Morel N. (2004). Comparative study of theantihypertensive activity of *Marrubium vulgare* and of the dihydropyridine calcium antagonist amlodipine in spontaneously hypertensive rat. Clin. Exp. Hypertens..

[4] Zaouali Y., Messaoud C., Ben Salah A., Boussaïd M. (2005). Oil composition variability among populations in relationship with their ecological areas in Tunisian *Rosmarinus officinalis* L.. Flavour Fragr. J..

[5] Zaabat N., Hay A., Michalet S., Darbour N., Bayet C., Skandrani I., Chekir-Ghedira L., Akkal S., Dijoux-Franca M. (2011). Antioxidant and antigenotoxic properties of compounds isolated from *Marrubium deserti* de Noé. Food Chem. Toxicol..

[6] Pottier-Alapetite G. (1981). Flore de la Tunisie, Angiospermes dicotyledones.

[7] Kadri A., Zarai Z., Ahmed Békir A., Gharsallah N., Damak M., Gdoura R. (2011). Chemical composition and antioxidant activity of *Marrubium vulgare* L. essential oil from Tunisia. Afr. J. Biotechnol..

[8] Boudjelal A., Henchiri C., Siracusa L., Sari M., Ruberto G. (2012). Compositional analysis and in vivo anti-diabetic activity of wild Algerian *Marrubium vulgare* L. infusion. Fitoterapia.

[9] Hussain J., Ullah R., Khan A., Mabood F., Shah M.R., Al-Harrasi A., Gilani A.H. (2011). Antispasmodic and Ca++ antagonist potential of marrubiin, a labdane type diterpene from *Phlomis bracteosa*. J. Pharm. Res..

[10] Ortega-Ramirez L.A., Rodriguez-Garcia I., Leyva J.M., Cruz-Valenzuela M.R., Silva-Espinoza B.A., Gonzalez-Aguilar G.A., Siddiqui M.W., Ayala-Zavala J.F. (2014). Potential of Medicinal Plants as Antimicrobial and Antioxidant Agents in Food Industry: A Hypothesis. J. Food Sci..

[11] Yousefi K., Soraya H., Fathiazad F., Khorrami A., Hamedeyazdan S., Maleki-Dizaji N., Garjani A. (2013). Cardioprotective effect of methanolic extract of *Marrubium vulgare* L. on isoproterenol-induced acute myocardial infarction in rats. Indian J. Exp. Biol..

[12] Yildirim A.B., Karakas F.P., Turker A.U. (2013). In vitro antibacterial and antitumor activities of some medicinal plant extracts, growing in Turkey. Asian Pac. J. Trop. Med..

[13] Daoudi A., Aarab L., Abdel-Sattar E. (2013). Screening of immunomodulatory activity of total and protein extracts of some Moroccan medicinal plants. Toxicol. Ind. Health.

[14] Edziri H., Mastouri M., Aouni M., Verschaeve L. (2012). Polyphenols content, antioxidant and antiviral activities of leaf extracts of *Marrubium deserti* growing in Tunisia. S. Afr. J. Bot..

[15] Ahmed B., Masoodi M.H., Siddique A.H., Khan S. (2010). A new monoterpene acid from *Marrubium vulgare* with potential antihepatotoxic activity. Nat. Prod. Res..

[16] Stulzer H.K., Tagliari M.P., Zampirolo J.A., Cechinel V., Schlemper V. (2006). Antioedematogenic effect of marrubiin obtained from *Marrubium vulgare*. J. Ethnopharmacol..

[17] Pavela R. (2004). Insecticidal activity of certain medicinal plants. Fitoterapia.

[18] Baccelli C., Navarro I., Block S., Abad A., Morel N., Quetin-Leclercq J. (2007). Vasorelaxant activity of diterpenes from Croton zambesicus and synthetic trachylobanes and their structure-activity relationships. J. Nat. Prod..

[19] Ulukanli Z., Akkaya A. (2011). Antibacterial Activities of *Marrubium catariifolium* and *Phlomis pungens* Var. Hirta Grown Wild in Eastern Anatolia, Turkey. Int. J. Agric. Biol..

[20] Zarai Z., Kadri A., Ben Chobba I., Ben Mansour R., Bekir A., Mejdoub H., Gharsallah N. (2011). The in vitro evaluation of antibacterial, antifungal, and cytotoxic properties of *Marrubium vulgare* L. essential oil grown in Tunisia. Lipids Health Dis..

[21] Emberger L. (1966). Une classification biogeographique des climats. Recl. Trav. Lab. Bot. Géol. Zool. Fac. Sci. Univ. Montpel..

[22] Adams R. (2004). Identification of Essential Oil Components by Gas Chromatography/Quadrupole Mass Spectroscopy.

[23] Mau J.L., Chao G.R., Wu K.T. (2001). Antioxidant properties of methanolic extracts from several ear mushrooms. J. Agric. Food Chem..

[24] Djeridane A., Yousfi M., Nadjemi B., Boutassouma D., Stocker P., Vidal N. (2006). Antioxydant activity of some Algerian medicinal plant’s extracts containing phenolic compounds. Food Chem..

[25] SAS Institue Inc. (2006). SASSAS User’s Guide: SAS STAT, SAS BASIC. Version 6.2.

[26] Chappell J., Coates R.M. (2010). Natural Products Structural Diversity-I Secondary Metabolites: Organization and Biosynthesis. Comprehensive Natural Products II.

[27] Goodarzi S., Hadjiakhoondi A., Yassa N., Khanavi M., Tofighi Z. (2016). New Benzodioxole Compounds from the Root Extract of *Astrodaucus persicus*. Iran J. Pharm. Res..

[28] Gupta S.D., Rao G.B., Bommaka M.K., Raghavendra N.M., Aleti S. (2016). Eco-sustainable synthesis and biological evaluationof 2-phenyl 1,3-benzodioxole derivatives as, DNA binding and antibacterial agents. Arab. J. Chem..

[29] Rezgui M., Mabrouk B., Neng N., Nogueira J.M., Ben-Kaab L.B., Machado Araújo M.E. (2021). Evaluation of *Marrubium vulgare* GrowingWild in Tunisia for Its Potential as a Dietary Supplement. Foods.

[30] Morteza-Semnani K., Saeedi M. (2004). The Essential Oil Composition of *Marrubium astracanicum Jacq*. Iran. J. Essent..

[31] Khanavi M., Ghasemian L., Hosseiny Motlagh E., Hadjiakhoondi A., Shafiee A. (2005). Chemical composition of the essential oils of *Marrubium parviflorum* fisch. & C. A. Mey and *Marrubium vulgare* L. from Iran. Flavour Fragr. J..

[32] Asadipour A., Mehrabani M., Nazeri V., Tabarraii M. (2005). Composition of the essential oil of *Marrubium vulgare* L.. Pharm. Sci..

[33] Zawislak G. (2012). The Chemical Composition of the Essential Oil of *Marrubium Vulgare* L. from Poland. Farmacia.

[34] Nagy M., Svajdlenka E. (1998). Comparison of Essential Oils from *Marrubium vulgare* L. and *M. peregrinum* L.. J. Essent. Oil Res..

[35] Weel K.C.G., Venskutonis P.R., PukalsKas A., Gruzdiene D., Linssen J.P.H. (1999). Antioxidant activity of horehound (*Marrubium vulgare*) growing Lithuania. Eur. J. Lipid Sci. Technol..

[36] Mkaddem M., Boussaid M., Ben Fadhel N. (2006). Variability of Volatiles in Tunisian *Mentha pulegium* L. (Lamiaceae). J. Essent. Oil Res..

[37] Ben El Hadj Ali I., Guetat A., Boussaid M. (2012). Genetic diversity and structure of wild Tunisian *Thymus capitatus* (L.) Hoffm. et Link. (Lamiaceae) assessed using isozyme markers. Afr. J. Ecol..

[38] Nevo E. (1988). Genetic Diversity in Nature. Evol. Biol..

[39] Prober S.M., Brown A.H.D. (1994). Conservation of the Grassy White Box Woodlands: Population Genetics and Fragmentation of *Eucalyptus albens*. Conserv. Biol..

[40] Zaouali Y., Boussaid M. (2007). Isoenzyme markers and volatiles in Tunisian *Rosmarinus Officinalis* L. (Lamiaceae): A comparative analysis of population structure. Biochem. Syst. Ecol..

[41] Chouaieb H., Ayadi I., Zouari S., Fakhfakh N., Zaidi S., Zouari N. (2012). Effect of Phenological Stage and Geographical Location on Antioxidant Activities of Tunisian Horehound: *Marrubium vulgare* L. (Lamiaceae). J. Biol. Act. Prod..

[42] Boulila A., Sanaa A., Salem I.B., Rokbeni N., M’rabet Y., Hosni K., Fernandez X. (2015). Antioxidant properties and phenolic variation in wild populations of *Marrubium vulgare* L. (Lamiaceae). Ind. Crops Prod..

[43] Wink M., Carey D. (1994). Variability of quinolizidine alkaloid profiles of *Lupinus argenteus* (Fabaceae) from North America. Biochem. Syst. Ecol..

[44] Zaouali Y., Chograni H., Trimech R., Boussaid M. (2012). Genetic diversity and population structure among *Rosmarinus officinalis* L. (Lamiaceae) varieties: Var. *typicus Batt*. and var. troglodytorum Maire. based on multiple traits. Ind. Crops Prod..

[45] Fawole O.A., Ndhlala A.R., Amoo S.O., Finnie J.F., Van Staden J. (2009). Anti-inflammatory, and phytochemical properties of twelve medicinal plants used for treating gastro-intestinal ailments in South Africa. J. Ethnopharmacol..

[46] Fratianni F., Tucci M., De Palma M., Pepe R., Nazzaro F. (2007). Polyphenolic composition in different parts of some cultivars of globe artichoke (*Cynara cardunculus* L. varscolymus (L.) Fiori). Food Chem..

[47] Lisiewska Z., Kmiecik W., Korus A. (2006). Content of vitamin C, carotenoids, chlorophylls and polyphenols in green parts of dill (*Anethum graveolens* L.) depending on plant height. J. Food Comp. Analyse..

[48] Yokoi N., Fujiwara Y., Wang H.Y., Kitao M., Hayashi C., Someya T., Tanamori M., Oiso Y., Tajima N., Yamada Y. (2008). Identification, and functional analysis of CBLB mutation in type 1 diabetes. Biochem. Biophis. Res..

[49] Aćimović M., Jeremić K., Salaj N., Gavarić N., Kiprovski B., Sikora V., Zeremski T. (2020). *Marrubium vulgare* L.: A Phytochemical and Pharmacological Overview. Molecules.

[50] Matkowski A., Piotrowska M. (2006). Antioxidant, and free radical scavenging activities of some medicinal plants from the Lamiaceae. Fitoterapia.

[51] Ksouri R., Megdiche W., Falleh H., Trabelsi N., Boulaaba M., Smaoui A., Abdelly C. (2008). Influence of biological, environmental and technical factors on phenolic content and antioxidant activities of Tunisian halophytes. C. R. Biol..

[52] Amri B., Martino E., Vitulol F., Corana F., Ben-Kaâb L.B., Rui M., Rossi D., Mori M., Rossi S., Collina S. (2017). *Marrubium vulgare* L. Leave Extract: Phytochemical Composition, Antioxidant and Wound Healing Properties. Molecules.

[53] Stanković M.S. (2011). Total phenolic, flavonoid concentration and antioxidant activity of *Marrubium peregrinum* L. extracts. Kragujevac. J. Sci..

[54] Djahral A.B., Bordjiba O., Benkherara S. (2012). Activité antibactérienne des flavonoides d’une plante médicinale spontanée *Marrubium vulgare* L. de la région d’El Tarf (Nord-Est Algérien). Rev. Sci. Technol..

